# Vermittlung der Terminservicestellen Psychotherapie im Jahr 2019: Eine Auswertung differenziert nach Vermittlungsanliegen und KV-Region

**DOI:** 10.1007/s00103-023-03756-7

**Published:** 2023-08-18

**Authors:** Deborah Engesser, Lena Maier, Markus Schepers, Susanne Singer

**Affiliations:** 1 Institut für Medizinische Biometrie, Epidemiologie und Informatik (IMBEI), Abteilung Epidemiologie und Versorgungsforschung, Universitätsmedizin der Johannes Gutenberg-Universität Mainz, Rhabanusstr. 3, Turm A, 55118 Mainz, Deutschland; 2 Institut für Medizinische Biometrie, Epidemiologie und Informatik (IMBEI), Abteilung Biometrie, Universitätsmedizin der Johannes Gutenberg-Universität Mainz, Rhabanusstr. 3, Turm A, 55118 Mainz, Deutschland

**Keywords:** Psychotherapie, Versorgung, Terminservicestelle, Kassenärztliche Vereinigung, Reform, Psychotherapy, Healthcare, Appointment service, Association of Statutory Health Insurance Physicians, Reform

## Abstract

**Hintergrund:**

Eine Maßnahme des Versorgungsstärkungsgesetzes war die Vermittlung psychotherapeutischer Leistungen (psychotherapeutische Sprechstunde, Akutbehandlung und probatorische Sitzungen) durch die Terminservicestellen der Kassenärztlichen Vereinigungen (KVen). Wir untersuchten diese Vermittlung differenziert nach Vermittlungsanliegen sowie nach Stadtstaat versus Flächenland.

**Methoden:**

Die Anzahl der Anfragen und Terminvereinbarungen im Jahr 2019 wurde bei den KVen Deutschlands mittels Fragebogen erfragt. Die statistische Analyse bestand aus einer deskriptiven Auswertung und einem Kruskal-Wallis-Test.

**Ergebnisse:**

Daten zu Terminvereinbarungen lagen von 17 KVen und Informationen zu Anfragen von 16 KVen vor. Insgesamt wurden 134.578 Termine vereinbart. Die Spannweite von 193 bis 21.810 Terminvereinbarungen offenbarte deutliche Unterschiede zwischen den einzelnen KVen. Der Anteil der psychotherapeutischen Sprechstunde an allen psychotherapeutischen Terminvereinbarungen lag im Median bei 92 %. Pro KV konnten im Median 87 % der Anfragen nach psychotherapeutischen Sprechstunden (Spannweite 56–100 %), 96 % nach Akutbehandlungen (29–100 %) und 97 % nach probatorischen Sitzungen (27–100 %) vermittelt werden (je *n* = 16). Es zeigten sich geringe Unterschiede zwischen Stadtstaaten und Flächenländern bei der Vermittlung von Akutbehandlungen und probatorischen Sitzungen.

**Diskussion:**

Bei KVen sowohl in Stadtstaaten als auch in Flächenländern bestehen Defizite insbesondere bei der Vermittlung von Akutbehandlungen und probatorischen Sitzungen. Unsere Ergebnisse lassen keine Rückschlüsse auf den Vermittlungsradius oder die Wartezeit auf Termine zu.

## Abstract (en)

**Background:**

One provision of the Care Provision Strengthening Act in Germany was that psychotherapeutic appointments (psychotherapeutic consultation, acute care, and probatory sessions) are now arranged through the Association of Statutory Health Insurance Physicians organizations. We examined how many appointments were requested and given in 2019, differentiated by appointment type and by city state versus areal state organizations.

**Methods:**

The frequency of requests and appointments made in 2019 were collected from the Association of Statutory Health Insurance Physicians organizations in Germany using a questionnaire. Statistical analysis comprised descriptive evaluations and a Kruskal–Wallis test.

**Results:**

Data for appointments were available from 17 and information on requests from 16 Association of Statutory Health Insurance Physicians organizations. A total of 134,578 appointments were given. The number of granted appointments ranged from 193 to 21,810, revealing substantial differences between the individual organizations. The share of psychotherapeutic consultations among all psychotherapeutic appointments granted amounted to a median of 92%. Per organization, appointments were given for a median of 87% of requests for a psychotherapeutic consultation (range 56–100%), 96% for acute care requests (range 29–100%), and 97% for probatory session requests (range 27–100%) (*n* = 16, respectively). There were minor differences between city states and areal states in granting acute care and probatory sessions.

**Discussion:**

There are deficits both in city state and areal state Association of Statutory Health Insurance Physicians organizations in terms of granting acute care and probatory sessions. Our results do not allow conclusions about distance to the practices or waiting times.

## Hintergrund

Mithilfe des „[Gesetzes] zur Stärkung der Versorgung in der gesetzlichen Krankenversicherung“ [1] sowie der Reform der Psychotherapierichtlinie im Jahr 2017 sollte die psychotherapeutische Versorgungssituation verbessert werden [2]. Mit der Reform der Psychotherapierichtlinie wurden u. a. neue Versorgungselemente wie die psychotherapeutische Sprechstunde und die Akutbehandlung eingeführt. Psychotherapeutische Sprechstunden sind Erstgespräche, bei denen Patient*innen über Psychotherapie aufgeklärt und Indikationen für eine Psychotherapie geprüft werden [3]. Die Inanspruchnahme der psychotherapeutischen Sprechstunde ist Voraussetzung für eine weitere Behandlung, wobei Erwachsene bei einem/einer Psychotherapeut*in bis zu 3 50-minütige (oder 6 25-minütige) und Kinder und Jugendliche bis zu 5 50-minütige bzw. 10 25-minütige psychotherapeutische Sprechstunden wahrnehmen können [3]. Die Akutbehandlung umfasst bis zu 12 Einheiten à 50 min und soll eine kurzfristige Behandlung z. B. in Krisensituationen ermöglichen, da sie – im Gegensatz zur sogenannten Richtlinienpsychotherapie – zwar anzeige-, aber nicht antragspflichtig ist [3].

Die Kassenärztlichen Vereinigungen (KVen) wurden gesetzlich dazu verpflichtet, über Terminservicestellen (TSS) Termine für die neu eingeführte psychotherapeutische Sprechstunde und Akutbehandlung sowie probatorische Sitzungen zu vermitteln [1, 4]. Probatorische Sitzungen dienen der Vorbereitung einer Richtlinientherapie [3] und gehörten auch schon vor der Reform der Psychotherapierichtlinie zum bestehenden Versorgungsangebot.

Um die Terminvermittlung durch die TSS zu ermöglichen, sind an der vertragsärztlichen Versorgung teilnehmende Psychotherapeut*innen verpflichtet, Sprechstundentermine an die jeweilige KV zu melden [3]. Hierfür wird Psychotherapeut*innen empfohlen, ein Terminkontingent bereitzuhalten, das durch die TSS vergeben werden kann [5]. Die Meldung von Terminen zu Akutbehandlung und probatorischen Sitzungen, welche bis zu 4 Einheiten à 50 min umfassen können, ist in der Psychotherapierichtlinie nicht verpflichtend geregelt und erfolgt in Eigenregie der KVen. Dabei soll sichergestellt sein, dass Termine innerhalb der gesetzlich vorgegebenen Frist von 4 Wochen vergeben werden [1].

Viele durch die TSS vermittelten Termine sind psychotherapeutische Anliegen – so stieg der Anteil der Anfragen nach psychotherapeutischen Leistungen an allen berechtigten Vermittlungswünschen von 39 % im Jahr 2020 auf 43 % im Jahr 2021 [6, 7]. Dies erscheint plausibel vor dem Hintergrund einer 12-Monats-Prävalenz psychischer Erkrankungen von 28 % in der Gesamtbevölkerung in Deutschland [8]. Eine Umfrage der Kassenärztlichen Bundesvereinigung (KBV) aus dem Jahr 2019 ergab, dass 14 % der Befragten in den letzten 3 Jahren Hilfe bei „[seelischen Problemen]“ [9, S. 27] bedurften und 64 % von diesen eine*n Psychotherapeut*in aufsuchten.

Der jährliche TSS-Evaluationsbericht der KBV enthält Informationen zur Zusammensetzung der Anfragen nach Psychotherapeut*innen und deren Vermittlungsquote [6, 7]. Die Vermittlungsquote unterscheidet dabei nicht nach einzelnen KV-Regionen, obwohl die psychotherapeutische Versorgungssituation regional unterschiedlich ist: So schwankten die Wartezeiten auf eine Richtlinientherapie im Jahr 2018 zwischen 11,6 Wochen in Berlin und 25,4 Wochen in Thüringen [10]. Die Bundespsychotherapeutenkammer berichtet bei einer Stratifizierung nach Bundesland ebenfalls eine Differenz von insgesamt 10 Wochen bei der Wartezeit auf eine Richtlinienpsychotherapie [11]. Sogar innerhalb einer KV variieren die Wartezeiten regional, wie aktuelle Daten der KV Bayern zeigen [12]. Die KBV weist in den Berichten zudem auf eine regional unterschiedlich starke Nutzung der TSS-Psychotherapie hin [6, 7]. Rabe-Menssen et al. (2019) heben hervor, dass die Wartezeiten in KVen, deren Region einem Stadtstaat (Bremen, Berlin und Hamburg) entspricht (im Folgenden: Stadt-KVen), geringer seien als in den KVen, deren Region ein Flächenland ist ([10]; im Folgenden: Flächenland-KVen, dies schließt auch die KVen Nordrhein und Westfalen-Lippe ein, die je einen Teil des Flächenlands Nordrhein-Westfalen abdecken). Eine Unterscheidung nach KV-Regionen sowie nach Stadt- und Flächenland-KVen könnte aufzeigen, ob sich die regional unterschiedliche Versorgungssituation auch in der Vermittlungsleistung der TSS widerspiegelt.

Weiterhin wird in den KBV-Berichten nicht nach psychotherapeutischer Sprechstunde, Akutbehandlung oder probatorischen Sitzungen unterschieden. Es ist jedoch davon auszugehen, dass die Vermittlung einer psychotherapeutischen Sprechstunde leichter gelingt als die Vermittlung einer Akutbehandlung oder von probatorischen Sitzungen, da diese freie Behandlungskapazitäten bei Psychotherapeut*innen erfordern. Die Bundespsychotherapeutenkammer berichtet, dass Psychotherapeut*innen nach einem Erstgespräch meist keine Akutbehandlung anbieten können [11]. Eine Unterscheidung nach Vermittlungsanliegen könnte aufzeigen, ob auch für diese mehr Ressourcen bindenden Anfragen ausreichend Vermittlungskapazitäten bestehen.

Aufgrund des hohen Stellenwerts psychotherapeutischer Anfragen innerhalb der TSS sowie der Relevanz dieser Versorgungssparte in der öffentlichen Gesundheitsversorgung lohnt sich ein genauerer Blick auf die Vermittlung der TSS-Psychotherapie. Mittels einer detaillierten Betrachtung kann aufgezeigt werden, inwiefern ein zugleich niederschwelliger als auch kurzfristiger Zugang zur psychotherapeutischen Versorgung geschaffen werden konnte. Wir untersuchten in der vorliegenden Studie daher,

- wie viele Anfragen nach psychotherapeutischen Sprechstunden, Akutbehandlungen und probatorischen Sitzungen die KVen erhalten (*Vermittlungsaufkommen*),
- für wie viele Anfragen ein Termin vereinbart werden kann (*Vermittlungsquote*),
- ob sich die *Vermittlungsquote* zwischen den einzelnen KV-Regionen sowie Stadt- und Flächenland-KVen unterscheidet und
- ob sich die *Vermittlungsquote* zwischen psychotherapeutischer Sprechstunde, Akutbehandlung und probatorischer Sitzung unterscheidet.

## Methoden

### Studiendesign

Es handelt sich um eine Querschnittstudie auf Basis von Sekundärdaten. Diese wurde im Rahmen der vom Innovationsfonds finanzierten Studie „Evaluation der Psychotherapie-Strukturreform“ (PT-REFORM) durchgeführt.

### Datenerfassung

Alle 17 KVen in Deutschland erhielten im April 2020 postalisch und per E‑Mail einen Fragebogen. Mit diesem wurden die Anzahl der Anfragen und Terminvereinbarungen für psychotherapeutische Sprechstunden, Akutbehandlungen und probatorische Sitzungen für die Jahre 2018 und 2019 in aggregierter Form erfasst. Dabei wurde nicht zwischen Anfragen für Kinder und Jugendliche oder Erwachsene sowie zwischen ärztlichen oder psychologischen Psychotherapeut*innen unterschieden. Die Datenerfassung endete im Juni 2020.

### Statistische Analyse

Die Darstellung des *Vermittlungsaufkommens* erfolgte deskriptiv anhand der Häufigkeit von Anfragen und Terminvereinbarungen. Um die Anzahl der Anfragen in Bezug zu den verfügbaren Ressourcen in der psychotherapeutischen Versorgung zu setzen, wurde die Anzahl der Bedarfsplanungsgewichte pro KV herangezogen. Ein Bedarfsplanungsgewicht von 1 bildet dabei in etwa einen vollen Versorgungsauftrag ab („ist jedoch aufgrund der Besonderheiten des Zulassungsrechts und der Bedarfsplanung nicht damit gleichzusetzen“ [13, S. 5]). Für jede KV wurde die Anzahl der Anfragen durch die Anzahl der Bedarfsplanungsgewichte für ärztliche und psychologische Psychotherapeut*innen des Jahres 2019 [14] geteilt, um so die Anzahl der Anfragen pro Bedarfsplanungsgewicht zu ermitteln.

Für die *Vermittlungsquote* wurde für jede KV der Prozentsatz der Terminvereinbarungen an allen Anfragen gebildet. Die Berechnung erfolgte sowohl separat für psychotherapeutische Sprechstunden, Akutbehandlungen und probatorische Sitzungen als auch für die Summe aller Terminvereinbarungen an allen Anfragen. Mithilfe eines Kruskal-Wallis-Tests wurde geprüft, ob sich die *Vermittlungsquote* zwischen psychotherapeutischer Sprechstunde, Akutbehandlung und probatorischen Sitzungen statistisch unterscheidet. Das Delta zwischen den KVen wurde deskriptiv dargestellt. Die Auswertung erfolgte mithilfe der Software Stata (StataCorp., 2021. Stata Statistical Software: Release 2017. College Station, TX: StataCorp LLC, USA), die Abbildungen wurden mithilfe von Excel (Microsoft Corporation, 2016, Redmond, USA) erstellt.

## Ergebnisse

Da für das Jahr 2018 zum Teil keine Daten zur Vermittlung probatorischer Sitzungen vorlagen, wurden für die Auswertungen nur die Daten des Jahres 2019 verwendet.

Für die Anzahl der Terminvereinbarungen im Jahr 2019 lagen Daten aller KVen vor. Eine KV konnte keine Daten zur Anzahl der Anfragen in ihrer Region liefern, sodass hierfür nur Daten von 16 KVen vorlagen. Demzufolge konnte die Vermittlungsquote für 16 der 17 KVen berechnet werden.

### Vermittlungsaufkommen

#### Anzahl Anfragen und Terminvereinbarungen

Bei den 16 KVen gingen im Jahr 2019 insgesamt 151.677 Anfragen ein. Pro KV gingen im Median 9011 Anfragen nach psychotherapeutischen Sprechstunden, Akutbehandlungen und probatorischen Sitzungen ein (Tab. 1). Deutliche Unterschiede zwischen den einzelnen KV-Regionen zeigten sich in der Spannweite von 193 bis 31.424 Anfragen (Delta = 31.231). Der Interquartilsabstand betrug 2014 bis 14.366 Anfragen; dies bedeutet, dass 50 % der KVen zwischen 2014 und 14.366 Anfragen erhielten.

**Table 1:** 

**Vermittlungsaufkommen aller KVen**	**Anzahl KVen**	**Anzahl Anfragen bzw. Terminvereinbarungen**				
Anzahl Anfragen	16	151.677				
Anzahl Terminvereinbarungen	17	134.578				
**Vermittlungsaufkommen pro KV**	**Anzahl KVen**	**Mittelwert**	**SD**	**Median**	**Minimum**	**Maximum**
Anzahl Anfragen	16	9480	8680	9011	193	31.424
Anzahl Terminvereinbarungen	17	7916	6301	7501	193	21.810

*SD* Standardabweichung, *KV* Kassenärztliche Vereinigung

Alle 17 KVen vereinbarten im Jahr 2019 insgesamt 134.578 Termine. Im Median waren das pro KV 7501 vereinbarte Termine. Auch hier zeigte sich eine breite Spannweite von 193 bis 21.810 Terminvereinbarungen (Delta = 21.617); 50 % der KVen vereinbarten zwischen 2009 und 11.281 Termine.

**Figure 1:**
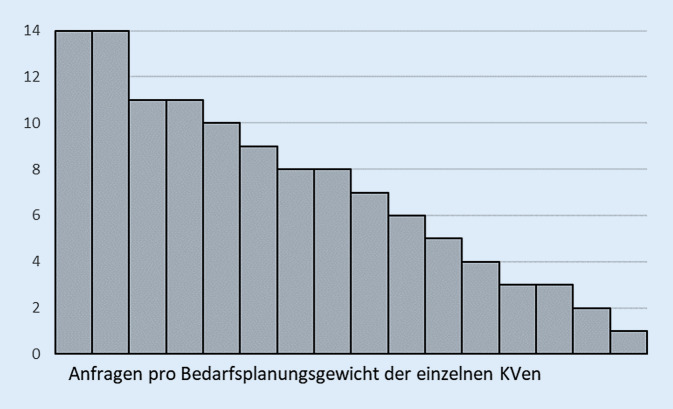

Das Verhältnis von Anfragen (Summe aus psychotherapeutischer Sprechstunde, Akutbehandlung und probatorischen Sitzungen) zu den Bedarfsplanungsgewichten zeigt, dass im Jahr 2019 pro KV auf ein Bedarfsplanungsgewicht im Median 8 Anfragen kamen, jedoch schwankt diese Zahl zwischen einer und 14 Anfragen (*n* = 16, Mittelwert 7, Standardabweichung 4 Anfragen, Abb. 1). Der Quotient aus allen Anfragen bei den 16 KVen im Jahr 2019 und allen Bedarfsplanungsgewichten zeigt, dass 7 Anfragen auf ein Bedarfsplanungsgewicht entfielen.

#### Zusammensetzung der Anfragen und Terminvereinbarungen

Von allen Anfragen, die im Jahr 2019 an die 16 KVen gerichtet wurden, entfielen 89 % auf psychotherapeutische Sprechstunden, 5 % auf Akutbehandlungen und 6 % auf probatorische Sitzungen (Tab. 2). 90 % der Terminvereinbarungen, die von allen 17 KVen getätigt wurden, beinhalteten einen Termin für eine psychotherapeutische Sprechstunde. Je 5 % der Terminvereinbarungen entfielen auf Akutbehandlungen oder probatorische Sitzungen.

**Table 2:** 

**Vermittlungsanliegen**	**Anzahl KVen**	**Anteil an allen Anfragen bzw. Terminvereinbarungen in %**				
*Zusammensetzung der Anfragen von 16 KVen*						
Psychotherapeutische Sprechstunde	16	89				
Akutbehandlung	16	5				
Probatorische Sitzung	16	6				
*Zusammensetzung der Terminvereinbarungen aller KVen*						
Psychotherapeutische Sprechstunde	17	90				
Akutbehandlung	17	5				
Probatorische Sitzung	17	5				
–	**Anzahl KVen**	**Mittelwert in %**	**SD in %**	**Median in %**	**Minimum in %**	**Maximum in %**
*Zusammensetzung der Anfragen pro KV*						
Psychotherapeutische Sprechstunde	16	90	5	89	80	98
Akutbehandlung	16	4	3	3	1	10
Probatorische Sitzung	16	6	4	4	1	13
*Zusammensetzung der Terminvereinbarungen pro KV*						
Psychotherapeutische Sprechstunde	17	91	5	92	82	98
Akutbehandlung	17	4	2	4	1	8
Probatorische Sitzung	17	5	4	4	1	13

*SD* Standardabweichung, *KV* Kassenärztliche Vereinigung

Betrachtet man die Zusammensetzung pro KV, so entfielen im Median 89 % der Anfragen und 92 % der Terminvereinbarungen auf die psychotherapeutische Sprechstunde, 3 % der Anfragen und 4 % der Terminvereinbarungen auf Akutbehandlungen sowie je 4 % auf probatorische Sitzungen (da es sich jeweils um den Median der Anteile jeder KV handelt, ergeben diese addiert nicht 100 %).

Der Anteil der Anfragen nach psychotherapeutischen Sprechstunden an allen Anfragen bei einer KV schwankte zwischen 80 % und 98 % (Delta = 18 %), der Anteil an Anfragen nach Akutbehandlungen lag zwischen 1 % und 10 % (Delta = 9 %). 1–13 % der Anfragenden forderten einen Termin für probatorische Sitzungen an (Delta = 12 %).

Die Zusammensetzung der Terminvereinbarungen variierte ebenfalls zwischen den KVen. 82–98 % der Termine wurden für psychotherapeutische Sprechstunden vereinbart (Delta = 16 %), 1–8 % für Akutbehandlungen (Delta = 7 %) sowie 1–13 % (Delta = 12 %) für probatorische Sitzungen.

### Vermittlungsquote

#### Vermittlungsquote von 16 KVen

81 % aller Anfragen bei 16 KVen nach einer psychotherapeutischen Sprechstunde im Jahr 2019 konnten vermittelt werden (Tab. 3). Bei der Akutbehandlung konnte für 62 % der Anfragen ein Termin vereinbart werden, bei probatorischen Sitzungen waren es 71 %.

**Table 3:** 

**Vermittlungsquoten Summe 16 KVen**						
**Vermittlungsanliegen**	**Anzahl KVen**	**Anteil aller Terminvereinbarungen an allen Anfragen in %**				
*Alle (datenliefernden) KVen*						
Psychotherapeutische Sprechstunde	16	81				
Akutbehandlung	16	62				
Probatorische Sitzung	16	71				
Gesamt	16	79				
*KV-Gebiet Stadtstaat*						
Psychotherapeutische Sprechstunde	3	83				
Akutbehandlung	3	76				
Probatorische Sitzung	3	62				
Gesamt	3	80				
*KV-Gebiet Flächenland*						
Psychotherapeutische Sprechstunde	13	80				
Akutbehandlung	13	58				
Probatorische Sitzung	13	75				
Gesamt	13	79				
**Vermittlungsquoten pro KV**						
**Vermittlungsanliegen**	**Anzahl KVen**	**Mittelwert in %**	**SD in %**	**Median in %**	**Minimum in %**	**Maximum in %**
*Alle (datenliefernden) KVen*						
Psychotherapeutische Sprechstunde	16	87	14	87	56	100
Akutbehandlung	16	84	21	96	29	100
Probatorische Sitzung	16	81	24	97	27	100
Gesamt	16	86	14	86	53	100
*KV-Gebiet Stadtstaat*						
Psychotherapeutische Sprechstunde	3	89	12	90	77	100
Akutbehandlung	3	83	15	77	73	100
Probatorische Sitzung	3	73	24	64	54	100
Gesamt	3	87	13	86	75	100
*KV-Gebiet Flächenland*						
Psychotherapeutische Sprechstunde	13	86	14	85	56	100
Akutbehandlung	13	84	23	99	29	100
Probatorische Sitzung	13	83	25	100	27	100
Gesamt	13	86	15	86	53	100

*SD* Standardabweichung, *KV* Kassenärztliche Vereinigung

Betrachtet man die Summe aller Anfragen bei den 16 KVen nach psychotherapeutischen Sprechstunden, Akutbehandlungen und probatorischen Sitzungen, so konnten 79 % der Anfragen im Jahr 2019 erfolgreich vermittelt werden.

#### Vermittlungsquoten einzelner KV-Regionen

Eine KV vermittelte im Median für 87 % der Anfragen nach einer psychotherapeutischen Sprechstunde einen Termin (Tab. 3). Bei der KV mit der geringsten Vermittlungsquote waren es 56 %, dagegen konnten bei anderen KVen 100 % der Anfragen vermittelt werden (Delta = 44 %).

Bei der Akutbehandlung fand im Median bei 96 % der Anfragen eine Terminvermittlung statt. Die KV mit der geringsten Vermittlungsquote konnte 29 % der Anfragen terminieren, jedoch konnten auch hier bei anderen KVen bis zu 100 % der Anfragen vermittelt werden (Delta = 71 %).

Ähnlich wie bei der Akutbehandlung konnte eine KV im Median für 97 % der Anfragen nach probatorischen Sitzungen einen Termin vereinbaren. Eine breite Spannweite zeigte sich auch hier mit Vermittlungsquoten von 27 % bis zu 100 % (Delta = 73 %).

Hinsichtlich der Vermittlungsquote der Summe der Anfragen nach psychotherapeutischen Sprechstunden, Akutbehandlungen und probatorischen Sitzungen ergab sich ein Median von 86 % erfolgreich vermittelter Anfragen pro KV mit einer Spannweite von 53–100 % (Delta = 47 %).

**Figure 2:**
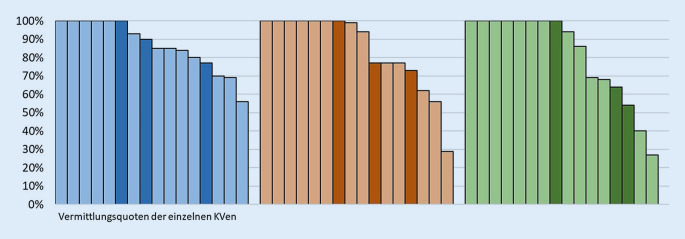

Beim Vergleich von Stadt-KVen mit den Flächenland-KVen zeigten sich Unterschiede bei Akutbehandlungen und probatorischen Sitzungen (Tab. 3; Abb. 2). Im Median konnten bei einer Stadt-KV 77 % der Anfragen nach Akutbehandlung vermittelt werden, während es bei einer Flächenland-KV im Median 99 % waren. Analog konnte bei einer Stadt-KV im Median für 64 % der Anfragen nach probatorischen Sitzungen ein Termin vermittelt werden, bei einer Flächenland-KV konnten im Median 100 % der Anfragen vermittelt werden. Die Minima von 29 % bzw. 27 % erfolgreich vermittelter Akutbehandlungen bzw. probatorischer Sitzungen zeigen jedoch, dass es auch bei den Flächenland-KVen vereinzelt erhebliche Defizite bei der Vermittlung gab.

Betrachtet man die Quoten für je alle Stadt- bzw. Flächenland-KVen zusammen, lassen sich bei der psychotherapeutischen Sprechstunde wenig Unterschiede erkennen – hier lagen die Vermittlungsquoten bei 83 % bzw. 80 % erfolgreich vermittelter Anfragen. Die Stadt-KVen konnten mit 76 % einen höheren Anteil der Anfragen nach einer Akutbehandlung vermitteln als die Flächenland-KVen mit 58 %. Dagegen konnten die Flächenland-KVen 75 % aller Anfragen nach probatorischen Sitzungen vermitteln, während es bei den Stadt-KVen 62 % waren.

#### Vermittlungsquote nach Vermittlungsanliegen

Ein Kruskal-Wallis-Test zeigte keinen signifikanten Unterschied zwischen der Vermittlungsquote von psychotherapeutischer Sprechstunde, Akutbehandlung und probatorischen Sitzungen (*n* = 16; χ2 = 0,02; *p* = 0,99).

## Diskussion

Wir gingen in dieser Studie der Frage nach, wie hoch das Vermittlungsaufkommen und die Vermittlungsquote bei der TSS-Psychotherapie der KVen sind. Dabei zeigte sich, dass sowohl die Anzahl der Anfragen und Terminvereinbarungen als auch der Vermittlungserfolg zwischen den einzelnen KV-Regionen heterogen sind. Es werden mehrheitlich psychotherapeutische Sprechstunden vermittelt.

Unsere Ergebnisse zeigten, dass im Jahr 2019 für 79 % aller an die KVen gerichteten Anfragen ein Termin vereinbart werden konnte. Diese Vermittlungsquote von 79 % basiert auf Daten von 16 der 17 KVen und bildet daher eine Annäherung an die Vermittlungsquote aller 17 KVen.

In den TSS-Evaluationsberichten der KBV wird die Vermittlungsquote für psychotherapeutische Anfragen auf 80 % im Jahr 2020 und 74 % im Jahr 2021 beziffert [6, 7]. Laut unseren Daten wurden im Jahr 2019 bei allen 17 KVen insgesamt 134.578 Termine vermittelt; die KBV berichtet von 175.319 fristgerechten Vermittlungen im Jahr 2020 und 235.390 im Jahr 2021 [6, 7]. Die Anzahl der vermittelten Termine stieg also, trotzdem konnte zwischen 2019 und 2020 eine gleichbleibende Vermittlungsquote erreicht werden, während 2021 weniger Termine erfolgreich vermittelt wurden. Die KBV begründet den allgemein verstärkten Andrang auf die TSS durch ein breiteres Aufgabenspektrum der TSS durch die COVID-19-Pandemie sowie durch eine verstärkte Medienpräsenz der TSS [6, 7]. Beim Bekanntheitsgrad der TSS als Erklärungsansatz ist zu berücksichtigen, dass die Vermittlung psychotherapeutischer Anliegen erst nach der Psychotherapiestrukturreform 2017 zur bereits bestehenden Facharztvermittlung durch die TSS hinzukam [1] und der Bekanntheitsgrad der TSS laut einer Befragung der KBV von 2016 zu 2019 eher abnahm [9]. Zunehmende psychische Belastungen durch die COVID-19-Pandemie [15–17] könnten erklären, warum sich laut unseren Daten im Jahr 2019 weniger Menschen an die TSS wandten als in den TSS-Evaluationsberichten der KBV für 2020 und 2021. Dass die Vermittlungsquote trotz steigenden Andrangs nahezu gleichblieb, erweckt den Anschein, dass der verstärkte Andrang im Jahr 2020 „aufgefangen werden“ konnte – es bleibt jedoch offen, ob Anfragende weitere Anfahrtswege in Kauf nehmen mussten oder ob sich die Wartezeiten auf die vermittelten Termine veränderten. Für die KV Bayern liegen Daten zu Wartezeiten sowohl für die Jahre 2017/2018 [10, 11] als auch für 2021 [12] vor. 2021 lag die mediane Zeitspanne zwischen psychotherapeutischer Sprechstunde und Beginn einer Richtlinientherapie bei 97 Tagen bzw. 13,9 Wochen [12]. Für die Jahre 2017/2018 ergibt sich eine Wartezeit zwischen Erstgespräch und Richtlinientherapie von 12,8 [10] bzw. 14 Wochen [11] (dies entspricht jeweils dem berichteten Mittelwert der Wartezeit zwischen Anfrage und Richtlinientherapie abzüglich des Mittelwerts der Wartezeit zwischen Anfrage und Erstgespräch). Auch wenn die Wartezeiten nicht direkt vergleichbar sind, deutet sich eine eher gleichbleibende Dauer an. Dies kann jedoch in anderen KVen variieren und lässt sich auch nicht auf alle Patientengruppen verallgemeinern, da die Wartezeit je nach Alter oder Wohnort stark schwankt [12]. Inwiefern sich die Wartezeit auf eine Akutbehandlung verändert haben könnte, lässt sich nicht bestimmen, da für 2021 keine Daten für die Akutbehandlung separat ausgewiesen wurden [12].

Limitationen unserer Studie sind vor allem bedingt durch fehlende Informationen zu den Vermittlungsdaten. So können wir keine Aussage darüber treffen, welche Personen (beispielsweise aus welcher Altersgruppe) sich an die TSS wandten oder für wen (beispielsweise Personen mit welchen Diagnosen) Termine vereinbart werden können. Für zustande gekommene Termine konnte die Wartezeit oder der Vermittlungsradius nicht bestimmt werden. Weiterhin wissen wir nicht, ob die Termine überhaupt zustande kamen (oder die Anfragenden diese aufgrund möglicher Anfahrtswege ausfallen ließen) und ob eine Anschlussbehandlung folgte. Hierbei sei hervorgehoben, dass trotz einer erfolgreichen Terminvermittlung Patient*innen oftmals nicht langfristig geholfen ist. Wenn auf psychotherapeutische Sprechstunden oder probatorische Sitzungen keine Behandlung erfolgen kann und Patient*innen sich immer wieder neu auf die Suche nach einem Therapieplatz begeben müssen, kann dies eine „erhebliche emotionale Belastung [darstellen]“ [18]. Unsere Daten erlauben keine Aussagen hinsichtlich der Versorgungssituation bei der Richtlinienpsychotherapie, da durch die TSS keine Therapieplätze für Richtlinientherapie vermittelt werden.

Zudem wurde nicht zwischen berechtigten und unberechtigten Anfragen unterschieden. Eine unberechtigte Anfrage liegt vor, wenn die Voraussetzungen für einen Vermittlungsvorgang nicht erfüllt sind (z. B. fehlende Kontaktdaten oder fehlende Diagnose, siehe auch [4, 6, 7]). Eine fehlende Terminvereinbarung für eine Anfrage und somit eine geringe Vermittlungsquote könnte somit sowohl durch fehlende Terminkapazitäten als auch durch eine fehlende Berechtigung der Anfrage begründet sein. Geringe Vermittlungsquoten sind aus unserer Sicht jedoch nicht allein durch fehlende Berechtigungen der Anfragen zu erklären und erscheinen vor dem Hintergrund der bereits andauernden angespannten Versorgungssituation mit langen Wartezeiten [10, 11] plausibel.

Die Aussagekraft unserer auf 16 KVen basierenden Vermittlungsquote schätzen wir aufgrund der nahezu erreichten Vollständigkeit als hoch ein.

Unsere Ergebnisse bestätigen die bereits berichtete Heterogenität der Versorgungssituation in den KV-Regionen [10] bzw. Bundesländern [11]. Hinsichtlich der Anzahl der Anfragen und Terminvereinbarungen lässt sich diese Heterogenität mit den unterschiedlichen Bevölkerungsdichten im Einzugsgebiet der einzelnen KVen erklären. So könnte eine geringe Anzahl von Anfragen auf eine geringe Personenanzahl im Einzugsgebiet einer KV mit Kenntnis der TSS-Psychotherapie zurückzuführen sein. Welcher Anteil der Patient*innen die TSS kennt, lässt sich aus unseren Daten jedoch nicht abschätzen. Eine heterogene Situation zeigte sich auch bei der Anzahl der Anfragen, die theoretisch auf ein Bedarfsplanungsgewicht entfallen. Hierbei ist jedoch zu beachten, dass unsere Daten nur die Anfragen bei der TSS abbilden. Patient*innen können sich auch direkt bei Psychotherapeut*innen melden, diese Anfragen sind den TSS aber nicht bekannt und wurden deshalb hier nicht berücksichtigt. Unterschiede zwischen den KVen zeigten sich also auf mehreren Ebenen: Sowohl hinsichtlich der Anfragen pro Bedarfsplanungsgewicht, hinsichtlich der Gesamtanzahl der Anfragen als auch hinsichtlich der Vermittlungsquoten. Da es auch aktuell „[regional unterschiedliche Rückmeldungen]“ zu Erreichbarkeit und Vermittlungskapazitäten der TSS gibt [18], ist zu vermuten, dass dies immer noch der Fall ist. Bei der Erklärung der unterschiedlichen Vermittlungsquoten muss eine unterschiedlich hohe Nachfrage in den einzelnen KV-Regionen [6, 7] berücksichtigt werden. Unterschiedlich lange Wartezeiten in einzelnen KV-Regionen bzw. Bundesländern [10, 11] lassen unterschiedlich viele freie Kapazitäten bei Psychotherapeut*innen vermuten. Der daraus resultierende Mangel an Vermittlungskapazitäten könnte ein Grund für die zum Teil geringen Vermittlungsquoten sein. Da die Meldung von freien Terminen für Akutbehandlungen und probatorische Sitzungen an die TSS in Eigenregie der einzelnen KVen erfolgt, könnte ein unterschiedlich striktes Vorgehen der KVen ebenfalls zu unterschiedlichen Vermittlungsquoten beitragen.

Vor allem bei der Vermittlung der Akutbehandlung und probatorischen Sitzungen bestehen deutliche Unterschiede zwischen den KVen. Auch gegenüber der psychotherapeutischen Sprechstunde bestehen tendenzielle Unterschiede, auch wenn sich diese nicht als statistisch signifikant erwiesen. Die medianen Vermittlungsquoten von 96 % bzw. 97 % erfolgreich vermittelter Akutbehandlungen bzw. probatorischer Sitzungen pro KV implizieren einen höheren Vermittlungserfolg als bei der psychotherapeutischen Sprechstunde mit einer medianen Vermittlungsquote von 87 %. Dies zeigt, dass bei den meisten KVen die Mehrheit der Anfragen erfolgreich vermittelt werden kann. Die breite Spannweite der Vermittlungsquoten pro KV (Minima 29 % bzw. 27 %) deutet jedoch darauf hin, dass Vermittlungsprobleme hinsichtlich der Akutbehandlung und probatorischen Sitzungen hauptsächlich bei einzelnen KVen bestehen. Dass Probleme insbesondere bei Akutbehandlung und probatorischen Sitzungen existieren, könnte damit erklärt werden, dass hier Kapazitäten für mindestens 12 Einheiten à 50 min bzw. eine Richtlinientherapie im Anschluss an die probatorischen Sitzungen erforderlich sind. Damit verbundene Probleme – vor allem bei probatorischen Sitzungen – zeigt auch der Verband der Ersatzkassen in einem aktuellen Forderungspapier auf [18]. Dagegen verlief die Vermittlung der psychotherapeutischen Sprechstunde homogener: Die mediane Vermittlungsquote von 87 % pro KV gleicht der Vermittlungsquote von 81 % aller 16 KVen (Anteil aller Terminvereinbarungen einer psychotherapeutischen Sprechstunde an allen Anfragen der 16 datenliefernden KVen). Auch hier gab es einzelne Ausreißer, die jedoch mit einem Minimum von 56 % weniger extrem ausfielen als bei Akutbehandlung und probatorischen Sitzungen.

Der Vergleich zwischen Stadt- und Flächenland-KVen zeigte, dass sowohl bei Stadt- als auch Flächenland-KVen Probleme bei solchen Vermittlungsleistungen bestanden, die mehr Kapazitäten in Anspruch nehmen (siehe oben). Dies zeigte sich bei den Stadt-KVen in mittlerem Ausmaß, während bei Flächenland-KVen eher vereinzelt erhebliche Defizite bestanden.

Analog zur Vermittlungsquote basiert die Zusammensetzung der Summe aller Anfragen auf 16 datenliefernden KVen und bildet somit eine Annäherung an die Zusammensetzung der Anfragen bei allen 17 KVen. Auch wenn sich die Anzahl der Anfragen zwischen 2019 und 2021 stark verändert hat (siehe oben), ist die Zusammensetzung über die Jahre vergleichbar geblieben. Die psychotherapeutische Sprechstunde dominiert mit 89 % im Jahr 2019 und laut KBV mit je 87 % in den Jahren 2020 und 2021 [6, 7] (aufsummiert für Kinder‑/Jugendlichen- und ärztliche/psychologische Psychotherapeut*innen). Ein Grund für die Dominanz der psychotherapeutischen Sprechstunde unter den Anfragen könnte sein, dass Patient*innen weiterführende Termine gezielt selbst vereinbaren (sofern eine Indikation für Psychotherapie besteht), beispielsweise auf Empfehlung der/des die Sprechstunde durchführenden Psychotherapeutin/Psychotherapeuten oder unter Berücksichtigung eigener Präferenzen, wie einem angemessenen Anfahrtsweg.

Die Dominanz der psychotherapeutischen Sprechstunde zeigt, dass die TSS hauptsächlich als „Türöffner“ zur psychotherapeutischen Versorgung fungiert. In dieser Hinsicht wird sie ihrer Rolle gerecht, da im Jahr 2019 bei allen 16 datenliefernden KVen mehr als die Hälfte, bei 12 KVen sogar mindestens 80 % der Anfragen nach psychotherapeutischen Sprechstunden vermittelt werden konnten. In der Anschlussversorgung spielt die TSS eher eine untergeordnete Rolle, da nur ein geringer Anteil der Vermittlungen auf Akutbehandlungen oder probatorische Sitzungen entfallen und keine Richtlinientherapie durch die TSS vermittelt wird. Hier zeigten sich zudem deutlichere Unterschiede zwischen den KVen als bei der psychotherapeutischen Sprechstunde. In einigen KV-Regionen kann die TSS durch erfolgreiche Vermittlung von Akutbehandlungen und probatorischen Sitzungen zur Anschlussversorgung beitragen, in einzelnen Regionen zeigten sich deutliche Defizite. Der Beitrag der TSS zur psychotherapeutischen Versorgung liegt also hauptsächlich im Angebot eines niedrigschwelligen Zugangs.

## Fazit

Unsere Daten zeigen in Kombination mit den Berichten der Kassenärztlichen Bundesvereinigung, dass seit 2019 mehr psychotherapeutische Anliegen an die Terminservicestellen (TSS) herangetragen werden. Die Differenzierung nach Regionen der Kassenärztlichen Vereinigungen (KVen) und Vermittlungsanliegen zeigte, dass es in einzelnen KV-Regionen Defizite hinsichtlich der Vermittlung von Akutbehandlung und probatorischen Sitzungen gibt. Bei der psychotherapeutischen Sprechstunde, welche den größten Anteil des Vermittlungsaufkommens ausmacht, konnten in 12 von 17 KV-Regionen mindestens 80 % der Anfragen vermittelt werden. Es ist fraglich, ob die TSS-Psychotherapie die Versorgungssituation verbessert hat. Einerseits bietet sie einen niedrigschwelligen Zugang, andererseits kann nicht gewährleistet werden, dass Anfragende eine angemessene psychotherapeutische Versorgung erhalten.
